# The Bnapus50K array: a quick and versatile genotyping tool for *Brassica napus* genomic breeding and research

**DOI:** 10.1093/g3journal/jkab241

**Published:** 2021-08-15

**Authors:** Qing Xiao, Huadong Wang, Nuan Song, Zewen Yu, Khan Imran, Weibo Xie, Shuqing Qiu, Fasong Zhou, Jing Wen, Cheng Dai, Chaozhi Ma, Jinxing Tu, Jinxiong Shen, Tingdong Fu, Bin Yi

**Affiliations:** 1 College of plant science and technology; National Key Laboratory of Crop Genetic Improvement; Huazhong Agricultural University, Wuhan, China, 430070; 2 Department of Biochemistry, School of Dental Medicine; University of Pennsylvania, Philadelphia, USA 19104-6303; 3 Greenfafa Institute of Novel Genechip R&D Co. Ltd., Wuhan, China 430010

**Keywords:** Bnapus50K, DNA array, *Brassica napus*, molecular breeding, single nucleotide polymorphisms (SNPs), genotyping tool, gene mapping

## Abstract

Rapeseed is a globally cultivated commercial crop, primarily grown for its oil. High-density single nucleotide polymorphism (SNP) arrays are widely used as a standard genotyping tool for rapeseed research, including for gene mapping, genome-wide association studies, germplasm resource analysis, and cluster analysis. Although considerable rapeseed genome sequencing data have been released, DNA arrays are still an attractive choice for providing additional genetic data in an era of high-throughput whole-genome sequencing. Here, we integrated re-sequencing DNA array data (32,216, 304 SNPs) from 505 inbred rapeseed lines, allowing us to develop a sensitive and efficient genotyping DNA array, Bnapus50K, with a more consistent genetic and physical distribution of probes. A total of 42,090 high-quality probes were filtered and synthesized, with an average distance between adjacent SNPs of 8 kb. To improve the practical application potential of this array in rapeseed breeding, we also added 1,618 functional probes related to important agronomic traits such as oil content, disease resistance, male sterility, and flowering time. The additional probes also included those specifically for detecting genetically modified material. These probes show a good detection efficiency and are therefore useful for gene mapping, along with crop variety improvement and identification. The novel Bnapus50K DNA array developed in this study could prove to be a quick and versatile genotyping tool for *B*. *napus* genomic breeding and research.

## Introduction

Rapeseed (*Brassica napus* L; AACC; 2*n* = 38) is the third widely grown oil crop in the world and is a rich source of healthy, edible oil. It is also grown to produce edible vegetables, succulents, and ornamental flowers. In addition, rapeseed can be used as a protein source for animal feedstuff and as a source of bioenergy. United States Department of Agriculture (USDA) data shows that the total global production of rapeseed oil and meal in 2018 was 71.94 and 38.79 million metric tons, respectively; this was second only to soybean production. The production of refined rapeseed oil was 27.46 million metric tons, with only palm and soybean oil exceeding this production value (USDA 2019, https://www.fas.usda.gov/). The breeding requirements of rapeseed are important for optimizing its production; therefore, there is a need to diversify these requirements given the versatility and worldwide use of this crop.

It is known that *B. napus* is an allopolyploid species that emerged during post-Neolithic speciation from a small number of natural hybridizations between the diploid progenitor species *B*. *rapa* (AA, 2*n* = 20) and *B*. *oleracea* (CC, 2*n* = 18) (Chalhoub *et al.* 2014). After approximately 700 years of domestication, *B. napus* can be divided into spring, semi-winter, and winter types (Zou *et al.* 2019).

The *B. napus* reference genome, Darmor-*bzh* (∼1.1 G), was first sequenced in 2014 by Chalhoub and his colleagues, providing rich genomic background knowledge for the species. Thereafter, other researchers annotated two winter-type cultivars, “Darmor-*bzh*” and “Tapidor,” using the same method and identified differences in gene numbers (Bayer *et al.* 2017). Compared with the winter type, the semi-winter type is believed to have undergone potential genomic introgression with *B. rapa*, leading to genetic differences in the control of vernalization and other traits (Sun *et al.* 2017; Zou *et al.* 2019; Song *et al.* 2020). Recently, several re-sequencing projects have provided a great deal of information regarding the genomic structure and genetic diversity of rapeseed (Bayer *et al.* 2017; Lu *et al.* 2019; Wu *et al.* 2019). There are huge differences among young *B. napus* varieties, and precise information regarding these differences can assist in genomic breeding.

Through the in-depth study of genetics and application of biotechnology, genomic breeding has emerged as a useful tool. A genomic breeding strategy can be effective in diversifying crop requirements over a relatively short timescale. Genomic breeding is based on genetic, functional, and phenotypic gene data, and it requires high-throughput detection of DNA polymorphisms for the selection of target and nontarget genes. Genomic breeding also provides genetic insight at the genome level according to breeding objectives, thus, revolutionizing the efficiency and accuracy of breeding selection (Varshney *et al.* 2005). The key to this technique is genome-wide marker-assisted selection; the existing high-throughput genotyping techniques include DNA sequencing (Davey *et al.* 2011) and DNA arrays (Gupta *et al.* 2008).

Next-generation sequencing (NGS), a well-known genotyping method, has the advantages of high throughput, flexibility, and efficiency. However, this method has some shortcomings; in particular, it is difficult to directly compare single nucleotide polymorphism (SNP) base genotyping data between different individuals in low-coverage sequencing. In addition, the processing of sequencing data requires expert knowledge of bioinformatics, which has limited applications (Bevan and Uauy, 2013). These shortcomings make it difficult to apply on a large scale to breeding, especially to molecular breeding strategies.

DNA arrays (commonly known as DNA chips) consist of a collection of microscopic DNA spots attached to a solid surface. They are used to measure the expression levels of large numbers of genes simultaneously, or to genotype multiple regions of a genome (Gunderson *et al.* 2005). Currently, the most mature SNP chip detection platforms are the Illumina Infinium and Affymetrix Axiom. Despite their similarity in format, size, and application, these two chip detection platforms differ greatly in probe length, hybridization strategy, and SNP detection strategy (Perkel, 2008). Fixed SNP genotyping arrays may be preferred to NGS technology, as they can achieve high throughput in a cost-effective manner and can be specifically designed to target high-value functional alleles for traits of interest. Although the density of SNPs on an array is typically lower than that of SNPs assayed by NGS, the selection of array-based SNPs can be optimized for particular breeding applications (Varshney *et al.* 2014; Rasheed *et al.* 2017).

Breeding chips have been successfully applied to multiple crops. For example, in rice, the Illumina Infinium Rice6K (Yu *et al.* 2014), Cornell_6K_Array_Infinium_Rice (Thomson *et al.* 2017), Affymetrix GeneChip Rice 44 K (McCouch *et al.* 2010), and Illumina Infinium RiceSNP50 (Chen *et al.* 2014) have been widely used in germplasm resource screening, variety authenticity and purity identification, and genetic background analysis of breeding materials. The Illumina Infinium maize SNP50 gene chip has previously been used to analyze two populations of maize recombinant inbred lines and high-density genetic linkage maps; as a result, 20,913 and 14,524 markers were obtained for each population, respectively (Ganal *et al.* 2011). In a study of the domestication history of soybean, the Affymetrix Axiom whole-genome SNP chip, NJAU 355 K SoySNP, was used to analyze 105 wild and 262 cultivar lines, showing that soybean cultivars originated from central and northern China (Wang *et al.* 2016). To construct a high-density genetic linkage map of wheat, four wheat populations were scanned using the 90 K Infinium iSelect SNP wheat chip, and 29,692 SNP markers were mapped to 21 chromosomes of hexaploid wheat (Wen *et al.* 2017). Furthermore, the wheat 660 K SNP array was recently shown to act as a substitute for six other arrays (Sun *et al.* 2020).

For rapeseed, an Illumina Infinium Brassica 60 K has been developed (Clarke *et al.* 2016). This has been widely utilized for gene mapping, genome-wide association study (GWAS) analysis, germplasm resource analysis, and evolution analysis (Pelc *et al.* 2015; Jan *et al.* 2016; Qian *et al.* 2016; Wei *et al.* 2016; Liu *et al.* 2016a, 2016b; Xu *et al.* 2016; Pele *et al.* 2017; Stein *et al.* 2017; Shen *et al.* 2018; Zhou *et al.* 2018; Zou *et al.* 2018). However, SNPs in Brassica 60 K were generated from genomic and transcriptome datasets and mapped to a pseudo *B*. *napus* genome derived from concatenating the genome sequences of *B. rapa* and *B. oleracea*, only 22,526 of 54,866 SNPs in the Brassica 60 K array are uniquely aligned on the Darmor-*bzh* genome, reducing the accuracy of the experimental results and increasing experimental cost (Xu *et al.* 2016).

## Materials and methods

### Plant materials, DNA extraction, and array experiment

All *B. napus* materials used in this study are detailed in Supplementary Table S1. DNA was extracted from *B. napus* leaves using the Tiangen DNAsecure Plant kit (Cat. #DP320-03), following the manufacturer’s protocol. Array assays were performed according to the Infinium assay standard protocol (Infinium HD Assay Ultra Protocol Guide, http://www.illumina.com/), using the HiScan scanner (Illumina Inc., San Diego, CA, USA).

### Array design

All probes were divided into three categories: Illumina Infinium Brassica 60 K high-quality probes (Clarke *et al.* 2016), sites selected from re-sequencing data, and functional probes (Supplementary Figure S1).

First, based on the chip detection results of the 523 varieties natural population (505 *B. napus* cultivars and 18 *B. rapa* cultivars), high-quality probes were screened according to the following principles: (1) call frequency (Calls/(No_Calls+Calls)) > 0.8; (2) cluster separation score (Cluster Sep) ≥ 0.15; (3) frequency of the minor allele (minor frequency) > 0.05; (4) a unique match on the Darmor-*bzh* genome (v4.1, *E*-value < 1e-5, Percent Identity > 85%); and (5) probes not located within 200 bp of each other. After screening, 17,758 probes remained.

Second, SNP markers were selected from whole-genome re-sequencing data of 505 *B. napus* cultivars(495 *B. napus* cultivars and inbred lines for 5× coverage, along with a further 10 varieties for 20× coverage) (Tang *et al.* 2020). After quality control of the re-sequencing data, we used BWA-MEM software (https://sourceforge.net/projects/bio-bwa/) to align them with the Darmor-*bzh* genome (v4.1). Subsequently, 32,216,304 SNPs were extracted from the re-sequencing data using GATK version 3.6-0-g89b7209 (Depristo *et al.* 2011) with the parameters of “-T HaplotypeCaller -allowPotentiallyMisencodedQuals -emitRefConfidence GVCF.” SNPs were then further selected for according to the following criteria: (1) presence of two alleles; (2) minor allele frequency (MAF) ≥ 0.05 and mapping quality ≥ 30; (3) the second allele of each SNP present in least 10 varieties; (4) no other SNPs within the 50 bp flanking the SNP site at both sides; and (5) 50 bp flanking sequences with no other genomic match ≥ 85%. A total of 286,921 SNPs remained after this step.

Third, to design an array suitable for a wide range of different research objectives and applications, the *B. napus* genome (Darmor-*bzh* v4.1) was split into 8,495 bins, with 100 kb per bin. Thereafter, the *r*^2^ value of linkage disequilibrium (LD) for any two SNPs located on the same bin was calculated and SNPs were grouped using a greedy algorithm, based on whether r^2^ ≥ 0.65 (Carlson *et al.* 2004). This allowed us to identify 75,826 SNP groups for the whole genome. SNPs within the same group were considered to provide similar information during genotyping. All 286,921 markers were submitted through the Illumina Design Tool to determine the assay design scores for each marker. One to five probes per 100 kb of genomic region were added, based on the probe density in the first part, and three probes were added per 150 kb of upstream and downstream quantitative trait locus (QTL) sites related to flowering, yield, oil quality, and stress resistance, as reported in the GWAS literature (Luo *et al.* 2015; Zhang *et al.* 2015, 2018; Gacek *et al.* 2016; Körber *et al.* 2016; Lu *et al.* 2016, 2017; Wei *et al.* 2016; Li *et al.* 2016a, 2016b; Liu *et al.* 2016b; Sun *et al.* 2016a, 2016b, 2016c; Wu *et al.* 2016; Qu *et al.* 2017; Tan *et al.* 2017; Wang *et al.* 2017; Rahaman *et al.* 2018; Raman *et al.* 2018; Wan *et al.* 2018; Chen *et al.* 2018b; Zhou *et al.* 2018). In total, 286,921 SNPs were screened according to the following criteria: (1) Illumina design score ≥ 0.6; (2) the highest MAF values; and (3) different bins within 100 kb. This resulted in 26,184 SNP sites being selected to design the probe sequence.

Finally, 1,765 specific probes were designed for functional fertility genes (genetic male sterility genes, cytoplasmic male sterility [CMS], and corresponding restorer genes), mitotype-specific sequences, and mitochondrial specific sequences (MSS; Heng *et al.* 2017), flowering time genes (Wu *et al.* 2019), self-incompatibility genes, disease resistance QTLs, oil content QTLs, and transgenic elements. Altogether, 45,707 probes were selected for manufacture by Illumina. A Python script was used during probe design.

### SNP marker analysis and cluster file construction

The *B. napus* array was used to genotype *B. napus* plants (Supplementary Table S1) following the protocols provided by Illumina (www.illumina.com). Illumina manufacturing processes were employed to successfully synthesize 42,090 markers (Supplementary Table S2), which were analyzed with respect to genotype clustering using GenomeStudio 2.0.4 (version 2015; Illumina Inc.) (Staaf *et al.* 2008). The cluster file was constructed using data from 192 previously genotyped *B. napus* samples, which included 144 cultivars, 27 parent lines, and 21 F_1_ plants (https://github.com/Sherry-DD/Bnapus50K-array).

Detection results were imported into the GenomeStudio software and the default diploid pattern was used for genotyping. Statistical analysis showed that SNP loci consisted of the following types: SNP loci with no detectable signal (Supplementary Figure 2A), SNP loci without polymorphism (Supplementary Figure 2, B and C), and SNP loci with polymorphism (Supplementary Figure 2, D–F). Polymorphic SNP loci genotyping results could be classified as one of four types. The first type correctly classified the same SNP into the three genotypes, AA, AB, and BB (Supplementary Figure 2D). In the second type, the software recognized a high number of heterozygotes (Supplementary Figure 2E); this was due to the strong sequence similarity between the A and C genomes of *B*. *napus* and the fact that when both homologous loci are polymorphic, some SNPs will reflect homologous site blending by displaying cluster-like patterns. The third type was generated when the homologous loci were monomorphic, that is, five genotypes could be distinguished by their cluster (Supplementary Figure 2F; from left to right, the genotypes are AAAA, AAAB, AABB, ABBB, and BBBB). Finally, in the fourth type, the software could not genotype correctly (Supplementary Figure 2F). Our markers (41,658 markers on the *B*. *napus* nuclear genome) were further characterized into 9,474 polymorphic, 32,184 monomorphic, and 28,606 intergenomic markers. The default cluster file mistakenly recognized the P_1_ genotyping results as the hybrid F_1_ genotyping results; therefore, we needed to correct the cluster file based on the test materials to obtain the correct genotyping results (Supplementary Figure 2G).

### Graphic file construction

For most data, we used R/ggplot2 software for visualization, and TBtools to show the location of functional probes (Chen *et al.* 2018a).

## Results

### Characteristics of the Bnapus50K chip

To develop an effective SNP array for *B. napus*, more than 32,000,000 candidate SNPs from *B. napus* genome re-sequencing data (505 varieties from the natural population) were screened. A total of 26,184 high-quality probes were obtained for synthesis, with characteristics such as a unique matching position on the Darmor-*bzh* genome, high call frequency, good cluster typing in the population, and physical distance between the probes of more than 200 bp. Furthermore, probes with MAFs of less than 0.05 were filtered out to ensure that our chosen probes are suitable for used in a wide range of research and breeding programs.

Usually, SNPs within genic regions have greater potential to affect gene function than do intergenic regions. In this study, 30% of the SNPs were located in coding regions(exon regions and intron regions), 16% of the SNPs were located in no coding regions(1 kb promoter region and 3’UTR) (Supplementary Figure S3C). All these probes were distributed evenly along the whole Darmor-*bzh* genome (Supplementary Figure S3A), and the average distance between adjacent SNPs on the Bnapus50K array was 8 kb, with only 0.7% of the SNP gaps being greater than 100 kb (Supplementary Figure S3B).

### Automated genotype calling through cluster definition

The data generated by the Illumina Infinium platform were analyzed using GenomeStudio software, whose principle is to score each SNP genotype as AA, AB, or BB if there are three separated signal clusters (www.illumina.com). Therefore, by developing robust cluster files, the most efficient high-throughput applications can be achieved for Illumina arrays. However, the software’s default cluster file is based on diploid humans; thus, adjustment is needed to obtain a suitable cluster file for rapeseed.

**Figure 1:**
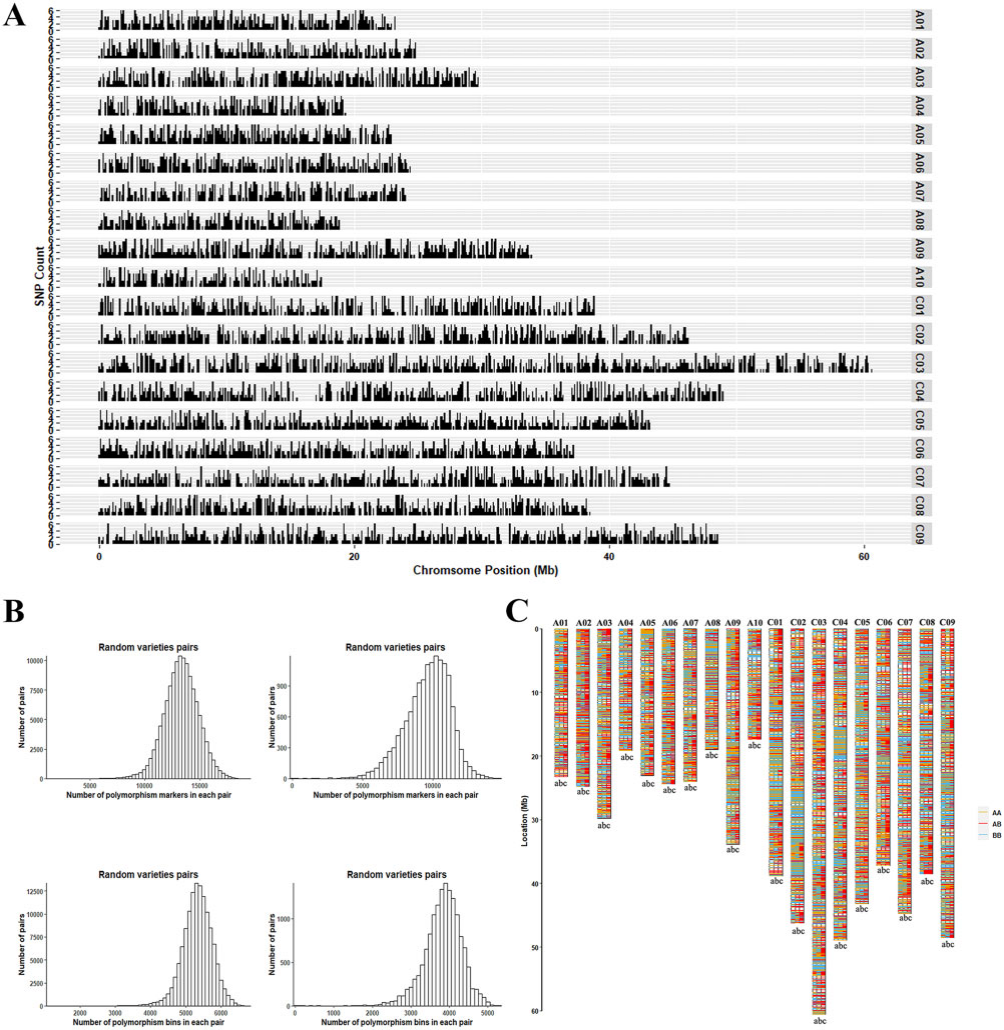
Characteristics of the high-quality Bnapus50K chip probes. (A) Genome-specificity markers distribution along the entire *B. napus* genome (Darmor-*bzh* v4.1). The markers were counts in each 100 kb regions. (B) Simulation calculation polymorphism markers between varieties (505 lines), based on whole-genome re-sequencing data (top left). Polymorphism markers between varieties (171 lines), based on Bnapus50K array genotyping (top right). Simulation calculation polymorphism bins between varieties (505 lines), based on whole-genome re-sequencing data (bottom left). Genomic polymorphisms between varieties (171 lines), based on Bnapus50K array genotyping (bottom right). (C) Genotype differences between P4, 11-997, and the offspring, F_1_. From left to right, the genotype distribution of P4 (“a” bars), 11-997 (“b” bars), and F_1_ (“c” bars) on the chromosome. The yellow line represents homozygous genotype AA, the blue line represents homozygous genotype BB, and the red line represents heterozygous genotype AB.

In this study, 144 cultivar lines, 27 parent lines, and their corresponding 21 F_1_ combinations were used as test materials for the Bnapus50K Illumina detection platform (Supplementary Tables S3 and S4; Supplementary Figure S4). Subsequently, we constructed a cluster file containing 28,509 high-quality probes. These were obtained by removing SNP sites that could not detect signals (5,445) or which had poor genotyping ability (8,136; could not distinguish between heterozygosity and homozygosity). There were 22,355 high-quality monomorphic markers, 19,752 of which were located on chromosomes (Figure 1A; Supplementary Table S5).

To validate the genotyping accuracy of the Bnapus50K array further, we randomly selected a set of crosses to observe the distribution of their genotypes (Figure 1C). The results showed that the different regions on the two parents were heterozygous for the hybrid progeny F_1_, thereby confirming the hybridization process.

### Detection effect of the array

To test the repeatability of sample testing by Bnapus50K, we performed technical and biological replicates. First, we used the same DNA for genotyping on different chips; there were 166 different probes, accounting for 0.58% of the total number of high-quality probes, which is close to the 0.1% recommended by Illumina. Second, we tested seedlings from different sources on the same chip and found that 0.92% of the probes (262) were different. These findings confirmed the high reproducibility of this efficient genotyping array. To validate the genotyping accuracy, the reference rapeseed cultivar “Westar” was used to compare the SNP sites that could be detected by the high-quality probes on array with the re-sequencing results; the consistency between the two was 98.4%.

A reliable array should have characteristics suitable for a variety of detection needs; therefore, we investigated the polymorphism of the screened high-quality probes in 144 cultivar and 27 parent lines. After excluding functional probes, we identified 28,156 high-quality probes (GenTrain score > 0.5) for analysis. We then examined whether the polymorphisms of these high-quality probes in the 171 lines were similar to the results obtained in previous simulations. We, thus, calculated the number of marker polymorphisms between the cultivar and parent lines (Figure 1B) and obtained the same results as the simulation. The number of polymorphism markers for most pairs was approximately 10,000; 68.31% of the pairs had more than 9,000 polymorphic markers. The distribution of polymorphic markers on the genome also matched that of the simulation (Figure 1B). Our results showed that 56.47% of the cultivar-parent pairs could detect approximately 45% of the genomic segment variation. That is, the polymorphic probes on the genome were uniformly distributed, indicating that our findings are in line with what was expected.

### Functional gene probes in the Bnapus50K array

**Figure 2:**
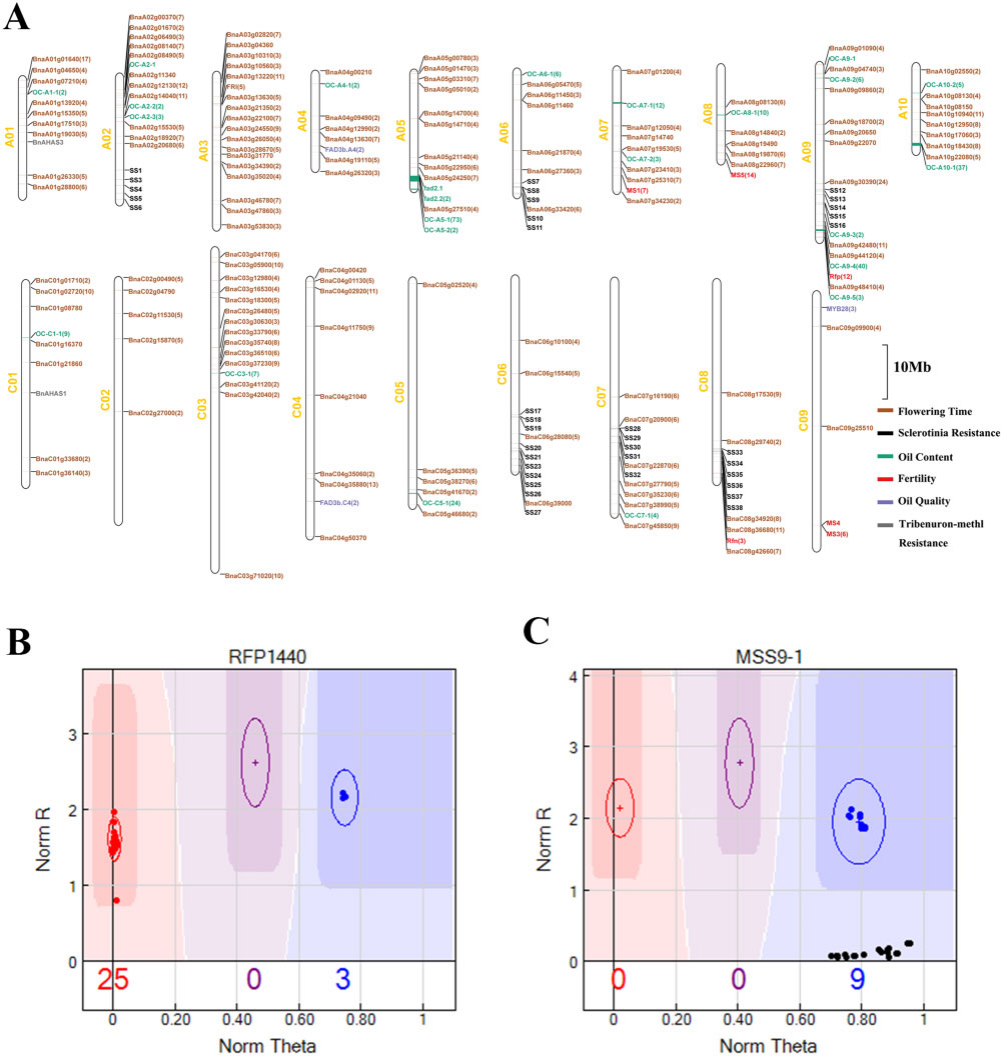
Characteristics of the functional probes. (A) Distribution pattern of functional gene or QTL probes. The number in parentheses indicates the number of probes synthesized by a given gene or QTL; no number in parentheses indicates that only one probe was synthesized by the gene or QTL. (B) The genotyping results for the cloned gene *Rfp* using the probe RFP1440. (C) Genotyping results for the cytoplasmic detection probe MSS9-1.

To expand the scope of its application, functional gene probes were added into the Bnapus50K array (Figure 2A; Supplementary Table S6). These functional gene probes were related to important agronomic traits such as oil content, disease resistance, male sterility, and flowering time. Probes related to oil content, flowering time, and disease resistance were designed based on the candidate genes obtained from GWAS analysis, while probes related to male sterility were designed based on the relevant cloned restorer gene and its alleles. Likewise, probes for self-incompatible genes were designed based on haplotype sequences in all Cruciferae species. The detection result of probes for the *Rfp* restorer (Figure 2B) and *pol* CMS (Figure 2C) genes showed that the Bnapus50K array is an effective genotyping tool.

### Bnapus50K array applications in crop improvement and identification

**Figure 3:**
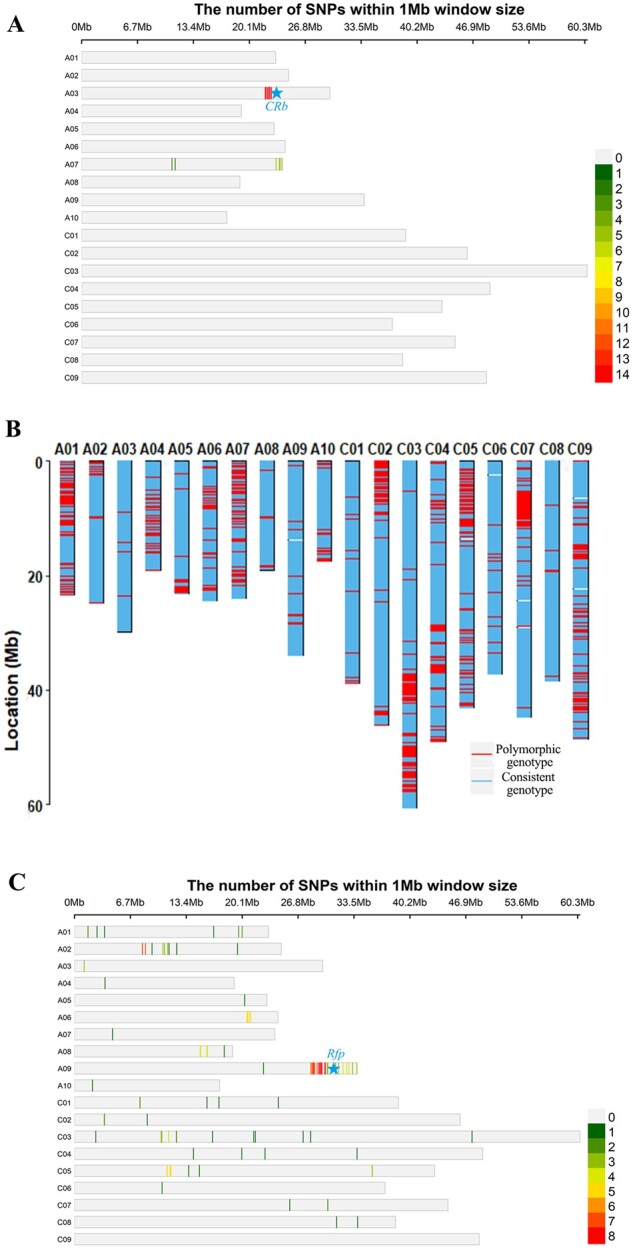
Bnapus50K array applications. (A) Polymorphism loci of clubroot resistant variety “B409R” and the recipient parent “B409.” The lines represent the differential probe numbers within a 1 Mb window size. The blue star represents the resistance gene *CRb*. (B) Detection results for two varieties with a pedigree relationship using the Bnapus50K array. The blue line represents the consistent genotype between “ZS9” and “ZS11,” and the red lines represent the polymorphic genotype between “ZS9” and “ZS11.” (C) Application of the Bnapus50K array in BSA via the mapping of the fertility restoring gene that controls *pol* CMS. The lines represent the differential number within a 1 Mb window size. The blue star represents the candidate gene *Rfp*.

An effective method for improving biotic or abiotic stress resistance is to breed corresponding varieties for each stress type. Here, two sets of genotype data were obtained from the clubroot-resistant line “B409R” and its recipient parent “B409.” Our results showed that the polymorphism probes were clustered around the disease resistance gene *CRb* (Figure 3A). Additionally, the four closest probes within the *CRb* locus (ChrA03: 23,693,689-24,031,219) were confined to a 215.96 kb interval on the Darmor-*bzh* genome, suggesting that the infiltration of targeted genome fragments can aid in improving resistance.

To conduct breed heterogeneity testing, the inbred lines “ZS9” (ZY821/84004 x ZS4) and “ZS11” (ZS9/2F10 x 26102) were genotyped. Our results showed that there were 5,268 (16.1%) polymorphism loci distributed in 2,176 genome bins (Figure 3B), confirming that “ZS9” and “ZS11” are two different lines. This indicates that the Bnapus50K array has great potential to be a useful tool in identifying the genetic relationships between *B. napus* varieties.

### Bnapus50K array applications in gene mapping

To test the suitability of the Bnapus50K array for gene mapping, A BC_2_F_1_ population constructed from “B409” (restorer) and “1318” (sterile) lines was used to map the fertility-restorer gene *Rfp* (previously mapped on chrA09 of Darmor-*bzh*) of “Polima” (*pol*) CMS (Liu *et al.* 2016c). Here, we combined bulked segregant analysis (BSA) with the Bnapus50K array; one fertile pool and one sterile pool were generated and genotyped using the Bnapus50K array. The genotyping results showed that the polymorphism probes for the two pools were primarily located on ChrA09 (Figure 3C). Notably, the genome fragments of ChrA09 that contained the cloned restorer gene *Rfp* exhibited a high density of polymorphism probes. Thus, the Bnapus50K array can be used to determine the location of target genes quickly and effectively.

### Bnapus50K array applications in detecting genetically modified organisms

**Figure 4:**
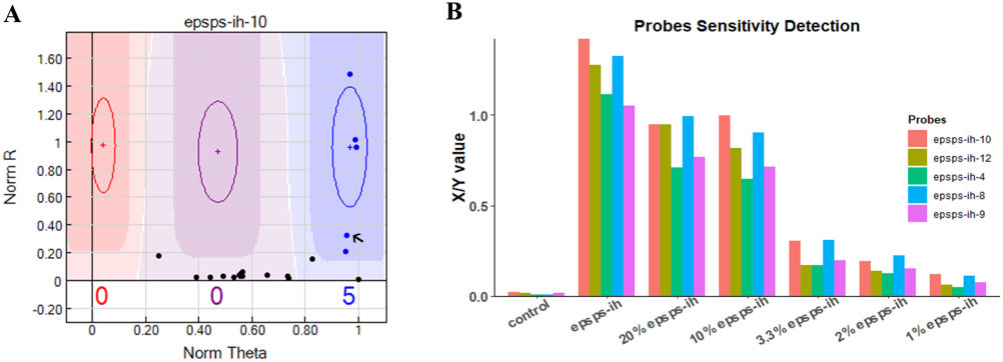
Detection sensitivity of transgenic element probes. (A) Genotyping results for the transgenic element detection probe epsps-ih-10; the arrow in the figure is the test result for negative and positive individual DNA at a ratio of 30:1. (B) Bar plots showing the detection sensitivity of the transgenic element probes.

Fourteen transgenic elements involved in eight independent transformation events were tested for probe quality (Supplementary Table S7) using nontransgenic varieties as negative controls. Furthermore, to explore the detection limits of the transgenic probes, we mixed the negative and positive individual DNA in proportions of 5:1, 10:1, 30:1, 50:1, and 100:1. Figure 4A illustrates the sensitivity and effective detection ability of these transgenic probes. As the content of negative DNA increases, the fluorescence signal intensity of the probes decrease significantly (Figure 4B). With 3.3% genetic modification, the X/Y value was greater than that of the negative control and could still be genotyped by the software (Supplementary Table S8). When the negative to positive ratio was 100:1, no significant difference was observed between the negative control and the test group.

## Discussion

### Bnapus50K as a practical genotyping tool

CMS is a widespread maternal genetic trait found in nearly 200 species; however, it can be restored by fertility genes in the nuclear genome (Feng *et al.* 2009; Liu *et al.* 2016c, 2017; Kim and Zhang, 2018; Wang *et al.* 2021). With the discovery and utilization of additional cytosolic types in *B. napus*, the efficient and rapid identification of these types are becoming increasingly important. As such, we designed cytoplasmic detection probes for *nap*, *cam*, *ole*, *pol*, *hau*, and *ogu* cytoplasm, based on MSS. Our results showed that these probes can clearly and specifically distinguish between different cytoplasm types, with four types of cytoplasmic probe being verified (Figure 2C; Supplementary Table S7).

Gene mapping is not only of great significance to scientific research but also has a direct effect on breeding improvements. Our results showed that probes with genotyping differences between fertile bulk and sterile bulk are mainly centered on the A09 chromosome, which is where the cloned restorer gene *Rfp* is located (Figure 3C). This indicates that the position interval of candidate genes can be obtained effectively using the Bnapus50K array. However, SNPs scattered in nontarget areas are generated owing to an insufficient number of mixed pool offspring. To obtain more accurate results, we recommend using three mixed offspring pools and one parental pool when combining BSA with the Bnapus50K array.

The application of genetically modified technology in crops has attracted controversy since its emergence. However, the cultivation of transgenic crops around the world has grown remarkably (Barragan-Ocana *et al.* 2019). The regulation of genetically modified rapeseed is important for its breeding success. Hence, an efficient and reliable array is required for transgene detection in the same way that detection probes for rice transgenic elements have been developed (and mentioned in international standard documents; Yu *et al.* 2014). Keeping this in mind, we developed an array with 291 probes synthesized for the efficient detection of genetically modified organisms (GMOs) and examined detection sensitivity based on the X/Y value given by GenomeStudio (Staaf et al. 2008). We found that plants subject to transgenic events could be efficiently genotyped in a highly sensitive and cost-effective manner using our innovative array. For future applications, we recommend using no more than 30 individuals per bulk to detect one or more positive individuals.

For our Bnapus50K array, we also focused on probe development for cloned functional genes; we added 1,618 functional probes specifically targeting high-interest traits for rapeseed breeding. Probes were designed to target sites related to quantitative traits such as flowering time, oil content, and stress resistance, which have the potential to improve the outcomes of genomic selective breeding. Moreover, specific probes for detecting genetically modified materials were designed to expand the range of Bnapus50K array applications. The addition of such probes will provide more intuitive data for rapeseed selection and assist in the process of rapeseed safety certification.

### Improving detection performance using Bnapus50K

The Illumina Infinium Brassica 60 K array is widely utilized in GWAS, germplasm resource, and evolution analysis, along with gene mapping (Pelc *et al.* 2015; Jan *et al.* 2016; Qian *et al.* 2016; Wei *et al.* 2016; Liu *et al.* 2016a, 2016b; Xu *et al.* 2016; Pele *et al.* 2017; Stein *et al.* 2017; Shen *et al.* 2018; Zhou *et al.* 2018; Zou *et al.* 2018). However, it was developed based on a pseudo-genome (Clarke *et al.* 2016), and only approximately 40% of its probes can be used in GWAS analysis (Xu *et al.* 2016).

Therefore, in this study, we took the LD value, probe density, and probe scoring value into consideration, which allowed an even distribution of SNPs on each chromosome. Compared with the probes in the Brassica 60 K chip, probes in the Bnapus50K array were more widely distributed across chromosomes (Supplementary Figure S3, A and B). Previous studies have confirmed that such consistency of physical and genetic distribution significantly improves the detection effect (Chen *et al.* 2014; Sun *et al.* 2020). The Bnapus50K array is, therefore, more suited to obtaining effective detection results in breeding or genomic research. Furthermore, this array comprises 1,618 functional probes, greatly improving its potential for use in a wide range of applications.

## Data availability

All data generated or analyzed during this study are included in this published article and its supplementary information files. Supplementary files are available at figshare: https://doi.org/10.25387/g3.14798436. The cluster file of Bnapus50K array is available at https://github.com/Sherry-DD/Bnapus50K-array.
